# Myxoma Virus Oncolytic Efficiency Can Be Enhanced Through Chemical or Genetic Disruption of the Actin Cytoskeleton

**DOI:** 10.1371/journal.pone.0084134

**Published:** 2013-12-31

**Authors:** Chad R. Irwin, Nicole A. Favis, Kate C. Agopsowicz, Mary M. Hitt, David H. Evans

**Affiliations:** 1 Department of Medical Microbiology & Immunology, University of Alberta, Edmonton Alberta, Canada; 2 Department of Oncology, University of Alberta, Edmonton Alberta, Canada; 3 Li Ka-Shing Institute of Virology, University of Alberta, Edmonton Alberta, Canada; Columbia University, United States of America

## Abstract

Myxoma virus (MYXV) is one of many animal viruses that exhibit oncolytic properties in transformed human cells. Compared to orthopoxviruses like vaccinia (VACV), MYXV spreads inefficiently, which could compromise its use in treating tumors and their associated metastases. The VACV F11 protein promotes virus exit and rapid spread by inhibiting Rho signalling, which results in a disruption of cortical actin. We have previously shown that although MYXV lacks an F11 homolog, the F11L gene can be introduced into MYXV promoting the spread of this *Leporipoxvirus* in natural host cells. Here we show that the F11-encoding (F11L^+^) MYXV strain replicates to higher levels in a number of human cancer cells. We also show that F11L^+^ MYXV induces better tumor control and prolonged survival of mice bearing MDA-MB-231 cancer cells. Furthermore, we show that this virus also spreads more efficiently from the site of growth in one injected tumor, to a second untreated tumor.

While we focused mostly on the use of a modified MYXV we were able to show that the effects of F11 on MYXV growth in cancer cells could be mimicked through the use of pharmacological inhibition or siRNA-mediated silencing of key regulators of cortical actin (RhoA, RhoC, mDia1, or LIMK2). These data suggest that it may be possible to increase the oncolytic efficacy of wild-type MYXV using chemical inhibitors of RhoA/C or their downstream targets. Furthermore, since all viruses must overcome barriers to exit posed by structures like cortical actin, these findings suggest that the oncolytic activity of other viruses may be enhanced through similar strategies.

## Introduction

An ideal oncolytic virus would not only spread efficiently throughout a tumor, but also travel rapidly to distant metastases, all while selectively killing cancer cells. One virus being pursued as an oncolytic virus is the *Leporipoxvirus* myxoma virus (MXYV). MYXV normally exhibits a very narrow host range limited to rabbits and hares and does not cause disease in humans. Nevertheless, it has been known for over 50 years that MYXV can replicate in cancerous human cells [1], [2] and over the past decade the molecular bases for this alteration in host tropism has begun to be understood. In particular, mutations that limit the activity of innate antiviral pathways (e.g. Type I IFN and TNF) as well as mutations that promote cellular proliferation (e.g. mutations in the AKT pathway) appear to enhance MYXV growth in transformed cells [3]–[8]. Genome-wide siRNA screens have further identified many additional human genes that collectively exert more subtle effects on MYXV growth [9].

While MYXV has shown promise as an oncolytic agent in a number of preclinical models, one potential problem with using MYXV as a therapeutic appears to be its limited ability to spread systemically in any environment aside from rabbits and hares. This was highlighted in 2005, where experimental gliomas were established in both hemispheres of the brains of nude mice. Injecting MYXV into one tumor caused its eradication, but no apparent effect on the second uninjected tumor was observed [10].

We have been examining how the cell exit and spread properties of MYXV differs from the *Orthopoxvirus* vaccinia (VACV) [11]. Despite both being poxviruses, in cell culture VACV disseminates much more rapidly than MYXV. One factor that contributes to efficient VACV spread is the formation of multiple types of infectious virus. Most VACV particles are surrounded by a single lipid bilayer and this form is called a mature virus (MV). MV are thought to be released upon cell lysis [12]. However, a fraction of MV can undergo additional maturation steps, which facilitate their exit prior to lysis [12]. These MV start by acquiring two additional lipid membranes, derived from either endosomes or the trans-Golgi network, along with several viral proteins. Known as wrapped virus (WV), these viruses then traffic along microtubules to the cell periphery, where the outermost membrane fuses with the cell membrane [12]. This releases a virus with one less lipid bilayer (known as an enveloped virus or EV) to the cell exterior. In addition to exiting cells prior to lysis, EV have additional benefits over MV, which aid in intra-host spread. EV are more resistant to neutralizing antibodies and complement than are MV [13], [14], and EV can also initiate actin projectile formation. Actin projectiles are formed when EV initiate an outside-in signalling cascade, which causes the polymerization of cellular actin at a position underneath the virus [12]. These virus-induced actin projectiles are thought to serve two purposes. First they probably provide a mechanical force that drives EV disassociation from the host cell [15]. Additionally, infected cells are thought to produce actin projectiles to repel incoming EV. This probably enhances virus spread by preventing the superinfection of already infected cells, and pushing away the EV until they encounter an uninfected cell [16]. Both viral and bacterial pathogens have been reported to manipulate the actin cytoskeleton to facilitate their spread (reviewed in [17], [18]), and this ability is an important *Orthopoxvirus* virulence factors (reviewed in [12], [19]).

While MYXV can form EV [20], in culture it forms far fewer actin projectiles than does VACV [11]. In trying to understand the genetics that underlie these differences we compared the genomes of MYXV and VACV for genes implicated in EV/actin projectile formation. We found MYXV homologs to most of these VACV genes, with the exception of a homolog of the VACV F11L gene. The F11 protein is known to bind to activated RhoA, and can not only inhibit its downstream signalling, but also recruits the GTPase activating protein Myosin-9A which promotes the conversion of RhoA back into an inactive signaling state [21]–[24]. By inhibiting RhoA signaling, VACV can perturb the structure of the cortical actin layer, an actin-rich region bordering the periphery of the cell, which provides attachment points and support for cellular structures like pseudopodia and the microtubular network [25]. F11 is known to disrupt this structure, which might otherwise interfere in forming contacts between the outermost membrane of a WV and the inner leaflet of the plasma membrane. This, in turn, promotes membrane fusion and EV release [21]–[23], [25], [26]. To test whether this natural difference between the two genera of viruses might have an effect on MYXV *versus* VACV spread, we have recently generated and characterized the *in vitro* properties of a strain of MYXV encoding the VACV F11L gene. This virus formed larger plaques, exhibited altered capacity to manipulate cellular actin, released more virus, and produced more actin projectiles [11].

These observations suggest that while MYXV naturally has a reduced capacity to manipulate the actin cytoskeleton, relative to VACV, it was still able to use F11 to aid its spread. This is of interest, as it has been shown that one can enhance the oncolytic activity of other viruses (e.g. VACV [27], [28] and reovirus [29]) by altering the efficiency of infection and spread. This led us to test whether increasing the efficiency of MYXV spread would also translate into making it a more effective oncolytic virus. Here we report that this recombinant strain of F11L-encoding MYXV grows better than control MYXV strains, in many different types of human cancer cells. Moreover, this increased growth is likely due to F11-mediated disruption of the actin cytoskeleton as illustrated by the use of pharmacological inhibitors of actin polymerization and with siRNAs targeting key genes in the RhoA-mDia signaling pathway. Lastly, we show that the MYXV F11L virus exhibits an enhanced capacity to inhibit tumor growth and a greater capacity to spread between tumors in a xenografted mouse tumor model. Together, these studies suggest that the oncolytic efficacy of MXYV (and perhaps other viruses) can be enhanced using genetic or pharmacological disruptors of the actin cytoskeleton.

## Results

### Manipulation of the actin cytoskeleton enhances MYXV growth

In a previous study, we showed that a strain of MYXV encoding the VACV F11L gene (F11L-mCh) spreads faster on rabbit and monkey cells in culture when compared with a control virus bearing the same mCherry insert in the non-essential MYXV M127L photolyase gene [30] but lacking F11L (ΔM127L-mCh). The enhanced spread can be attributed to the F11L-encoding virus having a superior capacity to modify the structure of the actin cytoskeleton, which causes a disruption of the cell's cortical actin. This, in turn, promotes the formation of actin projectiles and favours enhanced virus release [11]. We began this new study by investigating whether this phenotype could also be reproduced in human cells and whether we could mimic the phenotype through a combination of siRNA-mediated silencing and pharmacological inhibition of the actin cytoskeleton. For these initial studies, we used MDA-MB-231 mammary adenocarcinoma cells. These cells support an intermediate level of MYXV growth relative to other human cell types, and our lab previously used these cells in an siRNA-screen to identify cellular factors that facilitate MYXV growth [9]. MDA-MB-231 cells have also been widely used as a model for cancer research and there exists a wealth of biological data characterizing this cell line.

**Figure 1:**
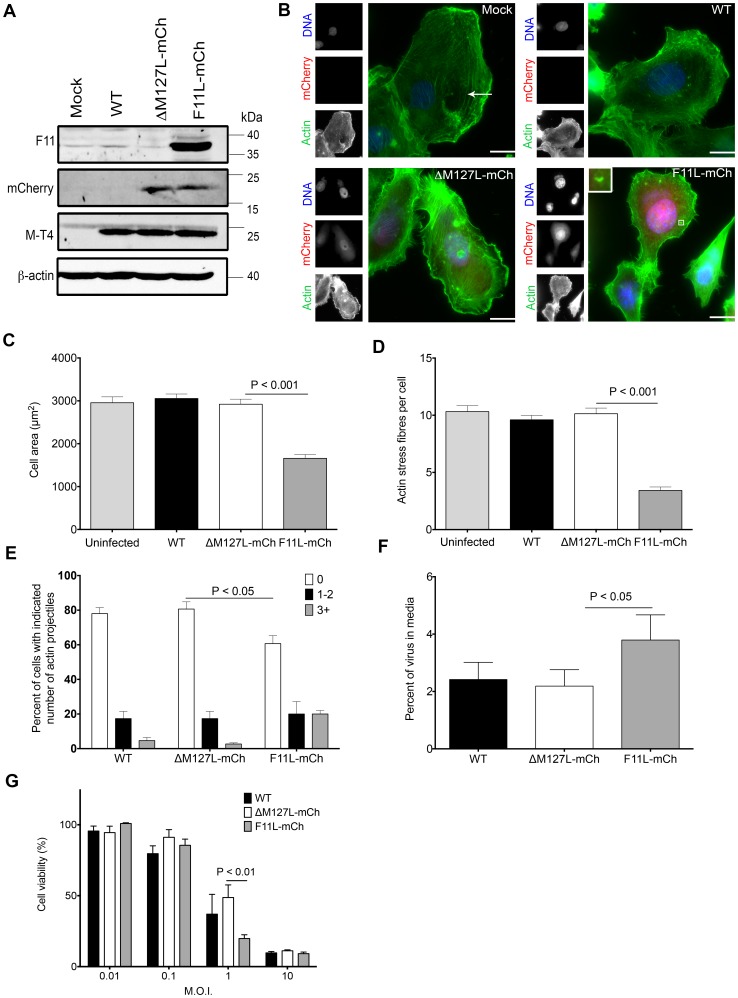
Effect of VACV F11 expression on MYXV properties in human MDA-MB-231 cells. **(A)** Western blot analysis of cells infected with the viruses used in this study. MDA-MB-231 cells were infected with the indicated viruses at MOI of 10, harvested 6 h post-infection, and western blotting was used to detect virus-encoded transgene products (F11 and mCherry), a MYXV gene product (M-T4) and a cell loading control (β-actin). **(B)** Fluorescence microscopy of MYXV-infected cells. MDA-MB-231 cells were infected with the indicated viruses at MOI of 10, and fixed 20 h post-infection. The cells were then stained and the actin, DNA, and virus-encoded mCherry were imaged at 60× magnification. Scale bar = 10 µm. To examine the effect of F11 on cell morphology, the cells were imaged as described in panel B, and the cell size and number of actin stress fibres measured at 8 h post-infection **(C+D)** or the number of actin projectiles measured at 20 h post-infection **(E)**. Actin projectiles are rare in MYXV-infected cells and one is shown enlarged in the bottom-right corner of panel B. An arrow points to a representative actin stress fibre in Panel B. The data shown were generated from three experiments, and 50 cells analyzed in each experiment (n = 150). **(F)** Virus released into the medium of infected cells. Cells were infected at MOI of 5, and 24 h later the amount of virus in the medium calculated as a percentage of total virus (media plus cell-associated virus). **(G)** Effect of virus on cell viability. Cells were infected at the indicated MOI, with each of three different virus strains, in 96-well plates. The cells were cultured for 96 h, and then the viability determined using Alamar blue dye. Viability is expressed as a percentage of that measured in uninfected cells. Panels C-G show the means ± S.E.M. from three independent experiments.

**Figure 2:**
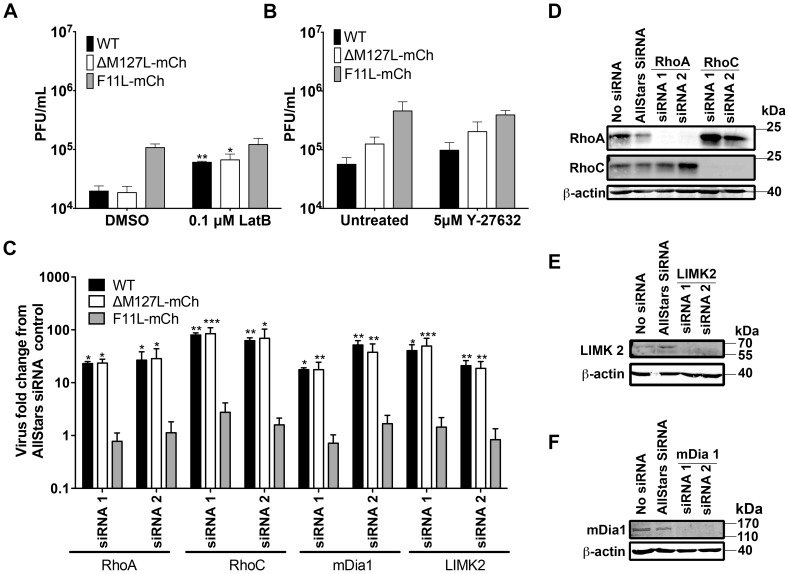
Effect of disrupting the actin cytoskeleton on MYXV growth in MDA-MB-231 cells. **(A)** Effect of latrunculin B on MYXV growth. MDA-MB-231 cells were infected with the indicated viruses at MOI = 0.1. The media was replaced 8 h later with fresh media containing 0.1 µM latrunculin B (or the DMSO solvent control), cultured 48 h, and the resulting virus harvested and titered. **(B)** Effect of ROCK inhibitor Y-27632 on MYXV growth. MDA-MB-231 cells were infected with the indicated viruses at MOI = 0.1. Four hours later the media was replaced with fresh media containing 0 µM or 5 µM Y-27632, cultured 48h, and the resulting virus harvested and titered. **(C)** Effect on MYXV growth of siRNA silencing of RhoA, RhoC, mDia1 or LIMK2. MDA-MB-231 cells were transfected with the indicated siRNAs for 48 h, and then infected with virus at MOI = 0.1. The viruses were harvested and titered 48 h later. For each siRNA treatment, we report the yield of virus measured relative to the amount of virus produced in cells that were transfected in parallel with a non-targeting AllStars siRNA control. **(D–F)** Western-blot analysis of proteins extracted from siRNA-treated cells. MDA-MB-231 cells were transfected with the indicated siRNAs and cultured for 48 h. The cells were then harvested, lysed, and western blotted using antibodies directed against RhoA and RhoC **(Panel D)**, mDia1 **(E)**, and LIMK2 **(F)**. β-actin was used as a loading control.

These cells can support the growth of our recombinant MYXV strains (**Figure 1A**), and in agreement with our previous observations in monkey and rabbit cells, infecting MDA-MB-231 cells with F11L^+^ MYXV produced alterations in the actin cytoskeleton, which were not caused by wild type (WT) or control (ΔM127L-mCh) MYXV (**Figure 1B**). At earlier times in infection (8 h), cells infected with the F11L-mCh virus were more rounded, had a smaller surface area (**Figure 1C**), and exhibited a ∼3-fold decrease in the number of central actin stress fibres when compared to cells infected with the control ΔM127L-mCh virus (3.4±0.3 fibres/cell *versus* 10.3±0.5 fibres/cell; P<0.001) (**Figure 1D**). F11 increases the VACV EVs exiting to the cell surface, where they can either disassociate or induce actin projectile formation [21], [22]. Similarly, we found that introducing F11L into MYXV increased both the percentage of MDA-MB-231 cells bearing actin projectiles and the amount of virus in media recovered from virus-infected cell cultures (**Figure 1E +F**). These F11-mediated alterations to the behaviour of MYXV-infected MDA-MB-231 cells were also were linked with an increase in cell death (**Figure 1G** **, S4**) although the effects varied by MOI. Furthermore, we observed that at low MOIs, F11 promoted greater growth of MYXV in MDA-MB-231 cells (**Figures 2** **,** **3** **and S3**).

**Figure 3:**
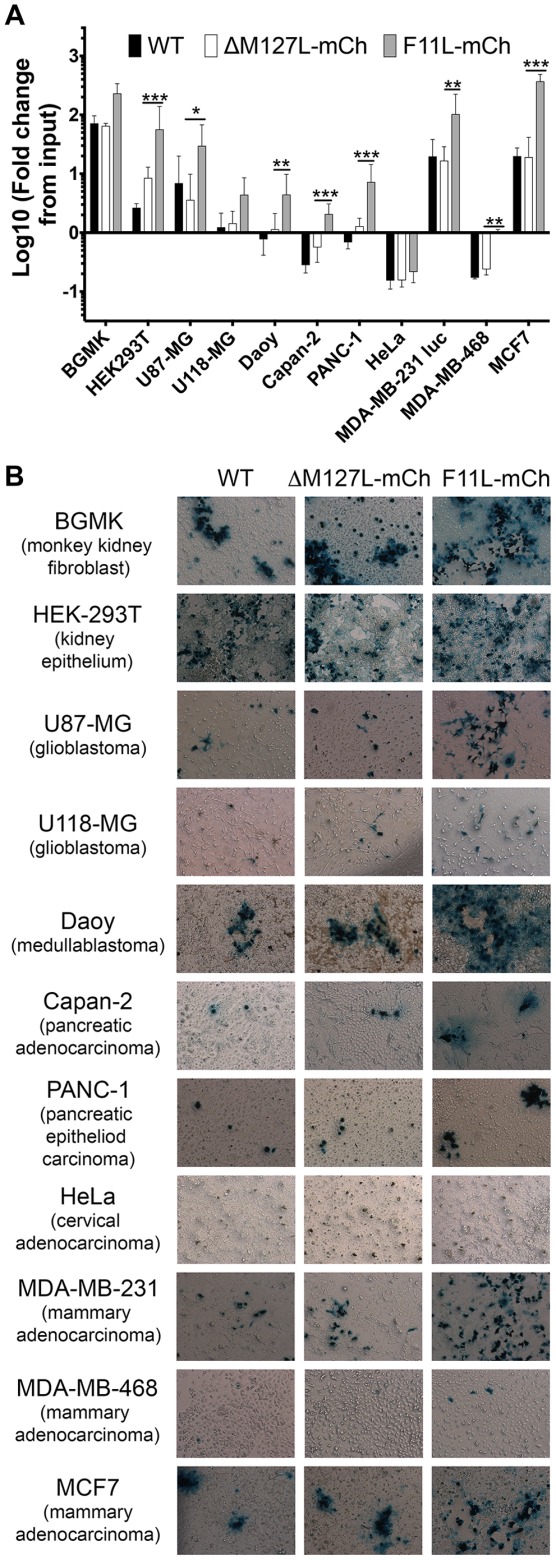
Effect of the F11L gene on MYXV growth in human cancer cells. **(A)** MYXV yields on different cell lines. The cells indicated were infected with WT, ΔM127L-mCh, or F11L-mCh viruses at MOI = 0.01. The viruses were harvested 72 h later, and titered on BGMK cells. For each cell and virus, we report the virus yield measured relative to the amount of input virus from three independent experiments (mean±S.E.M.) **(B)** MYXV plaque production on different cancer cells. The 11 different cell lines were infected with the indicated MYXV strains at MOI = 0.1. The cells were fixed four days later and the virus-encoded β-galactosidase activity detected using X-Gal. BGMK is a monkey cell line, the rest are of human origin.

Efficient VACV spread requires a balance between the disruption of cortical actin, to promote EV exit, and the availability of actin for other processes (e.g. actin projectiles). Important insights into these processes have been provided through the use of chemical inhibitors of actin dynamics like cytochalasin D [22], [31] and latrunculin B [22]. While high concentrations of latrunculin B is detrimental to VACV release, low concentrations are stimulatory and can overcome inhibitory effects of RhoA activation [22]. Since F11 seems to act similar to low concentrations of latrunculin B, we examined whether we could mimic the effects of F11 on MYXV growth by using this drug. MDA-MB-231 cells were infected with different viruses, cultured in the presence of 0.1 µM latrunculin B, and the resulting viruses titered. Latrunculin B treatment significantly increased the yields of WT and ΔM127L-mCh virus but had little effect on F11L-mCh infected cells (**Figure 2A and S** **1**). Similar to observations in VACV-infected cells [22], higher concentration of latrunculin B (1 µM) appeared to reverse this increase in growth (data not shown).

We next investigated whether disruption of RhoA signaling could also enhance MYXV growth. Active RhoA signals primarily to two effectors: ROCK (Rho-associated protein kinase) and mDia1 (also known as Dia1 or DIAPH1) [32]. While F11 binds to RhoA and presumably disrupts both pathways, the disruption of the mDia1 branch seems more important for virus release [21], [23], [25]. Consistent with observations with VACV we found that the use of the ROCK inhibitor Y-27632 had little effect on MYXV yields and actin projectile formation (**Figures 2B and S** **2**). This was despite induction of morphological rearrangements consistent with ROCK inhibition (**Figure S2**).

We also used siRNA-mediated gene silencing to see what effect inhibiting other elements of the Rho signaling pathway might have on MYXV growth. We found that depleting cells of RhoA or mDia1 enhanced the growth of WT and ΔM127L-mCh viruses relative to that in cells treated with a non-targeting siRNA control, but had little effect on the growth of F11L^+^ MYXV (**Figure 2C**). Western blots confirmed silencing by the two different siRNAs used to target each gene (**Figures 2D–F**). We also examined the effect of RhoC and LIMK2 depletion on MYXV growth. Although they display differences in their downstream effects on cell morphology and migration, RhoA and C both bind mDia1 and promote the formation of actin stress-fibres ([33], reviewed in [34]). In a recent genome-wide screen, we observed that LIMK2 knockdown appeared to promote MYXV growth in MDA-MB-231 cells (mDia1 was also identified in this screen). Activation of LIMK2, which is downstream of Rho and ROCK, has been shown to promote actin polymerization and actin stress fibre formation. This is due to phosphorylation and inactivation of cofilin by LIMK2, thus preventing actin depolymerization [35]. Furthermore, the actin cytoskeleton of MDA-MB-231 cells can be disrupted by siRNA knockdown of LIMK2 [36]. We observed that silencing of RhoC and LIMK2 also caused an increase in the growth of WT and ΔM127L-mCh viruses with little effect on F11L^+^ MYXV (**Figure 2C**). Together, these data suggest that MYXV growth can be enhanced by disruption of the actin cytoskeletal architecture using either pharmacological inhibitors, siRNA-dependent gene silencing, or by expressing a VACV-derived transgene (F11L).

### F11L^+^ MYXV grows to higher titers, and displays enhanced killing, in many kinds of human cancer cells

We previously noted that MYXV F11L-mCh grows to ∼5-fold higher titers in highly permissive monkey BGMK, and rabbit RK13 or SIRC cell lines. To test whether this was a general effect, we infected a panel of human cancer cells at low MOI (0.01 or 0.1) and measured the virus yields (**Figures 3A** **and S3**) or plaque morphology at 4 days (**Figure 3B**).The F11L^+^ MYXV grew better than the WT or ΔM127L-mCh viruses in nearly all of the cells tested. Interestingly, this effect varied greatly by cell line. In some cells (e.g. U118-MG and PANC-1) the effect of F11 on MYXV growth was similar to that seen in BGMKs, whereas in other cell lines (e.g. MCF7) the effect was much greater (∼30 fold). It should be noted that plaques observed in MCF7 cultures infected with the F11L virus appear unstained in the center because cells lysed by MYXV are no longer adherent. These data suggest that actin-based structures comprise a barrier to MYXV growth in a diversity of cancer cell types, and that perturbing these structures may serve as a means of increasing MYXV growth in cancer cells. We also observed that in some instances that cells infected with the F11L^+^ MYXV showed reduced viability suggesting that this virus also was capable of killing cancer cells better. This tended to be in situations where lower MOIs were used, but varied greatly by cell line (**Figure S4**).

Given that F11 appears to exert its effects on MYXV by inhibiting Rho signaling pathways, and that cancer cells express highly variable levels of the proteins regulating these pathways, we looked to see if there was any correlation between the abundance of these proteins and the effects of F11 on MYXV growth. While the levels of Rho signaling proteins varied greatly by cell-line, we could find no obvious correlation between the stimulatory effects of F11 on MYXV growth and the total levels of RhoA or RhoC, or of any of their downstream effectors (mDia1, ROCK1, Ezrin, LIMK2, or cofilin) (**Figure S5**). This may not be too surprising since F11 has been shown to bind active RhoA and can promote its inactivation through the recruitment of Myosin-9A [21], [24], and ordinary western blots cannot measure the degree of activation of these proteins.

### MYXV F11L-mCh is more effective at controlling tumor growth in xenografted animals

**Figure 4:**
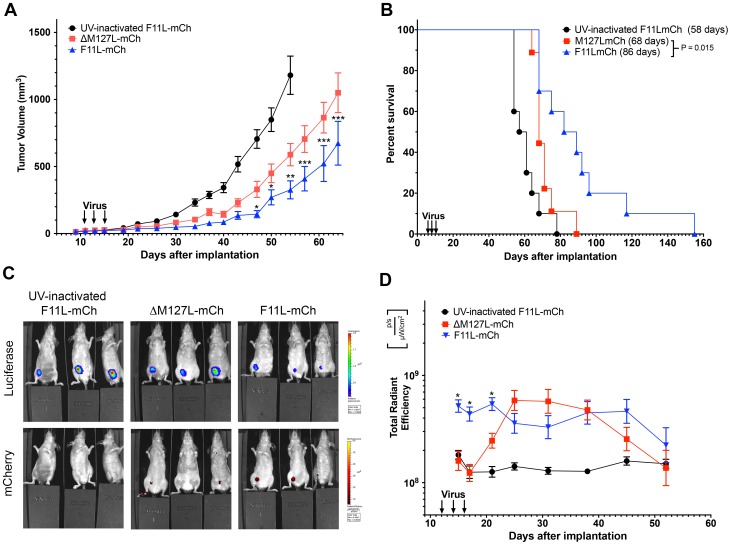
Effect of F11L^+^ MYXV on tumor progression and survival of NIH-III mice with xenografted MDA-MB-231 cells. Luciferase-tagged MDA-MB-231 cells were used to establish tumors in the mammary fat pads of NIH-III mice. Once the tumors were palpable, they were treated with three injections of the indicated mCherry-tagged MYXV (1×10^6^ pfu per injection) administered over a five-day period. For these experiments, 10 mice were used for each of the UV-inactivated and live F11L-mCh groups, and 9 mice were used for the ΔM127L-mCh virus. The statistics shown compare the live ΔM127L-mCh and F11L-mCh groups. **(A)** Tumor growth. Tumor size was measured twice-weekly using calipers.We report the mean tumor volume ± S.E.M for each group until the first mouse from each group reached endpoint. **(B)** Kaplan-Meier survival plot. The fraction of animals surviving at each time point is indicated as well as the median survival times for each cohort (inset). **(C)** Tumor and virus imaging. An IVIS Spectrum imager was also used to detect the tumor-encoded luciferase activity and virus-encoded mCherry protein. The example shows animals imaged at day 21, which is 10 days after the first virus injection. **(D)** Virus replication. The virus-encoded mCherry signal was used as a surrogate marker to estimate the level of virus replication. The figure shows the mean radiant efficiency ± S.E.M. Note that the signal from the UV-inactivated virus is essentially the same as the background signal in these experiments.

F11L's capacity to stimulate MYXV growth and enhance cell killing *in vitro*, led us to speculate that F11L^+^ MYXV might also be more effective than the wild-type virus at controlling tumor growth *in vivo*. To test this hypothesis, we established a xenografted tumor model using immunocompromised NIH-III mice bearing MDA-MB-231 tumors in the mammary fat pad. Once tumors were palpable, three intra-tumoral injections of virus (1×10^6^ pfu/injection) were administered, and tumor size monitored by caliper measurements. In comparison to mice treated with UV-inactivated virus, the tumors grew much slower in mice receiving either live ΔM127L-mCh or F11L-mCh virus. Furthermore, the tumors grew more slowly in mice treated with F11L^+^ MYXV compared with those receiving the live ΔM127L-mCh control virus, with the tumor volumes becoming significantly different by ∼45 days post-implantation (**Figure 4A**). This translated into a significant increase in the median survival in mice receiving F11L^+^ virus (86 days post-implantation *versus* 68 days for the ΔM127L-mCh virus; P = 0.015). In contrast, mice receiving UV-inactivated virus had a median survival of 59 days and this was also significantly different from the median survival in animals treated with the ΔM127L-mCh virus (P = 0.019) and F11L-mCh (P = 0.0001) (**Figure 4B**).

Although calipers provided the most accurate and quantitative way to monitor tumor growth, we also employed an IVIS imager to track the luciferase expressed by the xenografted cells and the fluorescent protein expressed from the replicating viruses (**Figure 4C and D**). We observed that significantly higher levels of the mCherry fluorescent signal appeared more rapidly in mice injected with the F11L^+^ virus and co-localized at all times with the tumor. The fluorescence in tumors treated with the ΔM127L-mCh control strain didn't “catch up” with the F11L-expressing strain until ∼10 days after imaging started (**Figure 4D**). The two viruses then continued to express similar levels of mCherry fluorescence for another ∼3 weeks. The F11L-mCh virus seemed to persist longer in the treated animals, with one mouse still exhibiting some mCherry signal (and detectible virus by plaque assay) when its tumor reached endpoint criteria 155 days after implanting the tumor. The luciferase signals also supported the caliper measurements. At any given time point the strongest luciferase signals were detected in animals treated with UV-inactivated virus, less so in ΔM127L-mCh treated tumors, and less again in animals treated with the F11L-mCh virus (**Figure 4C**).

**Figure 5:**
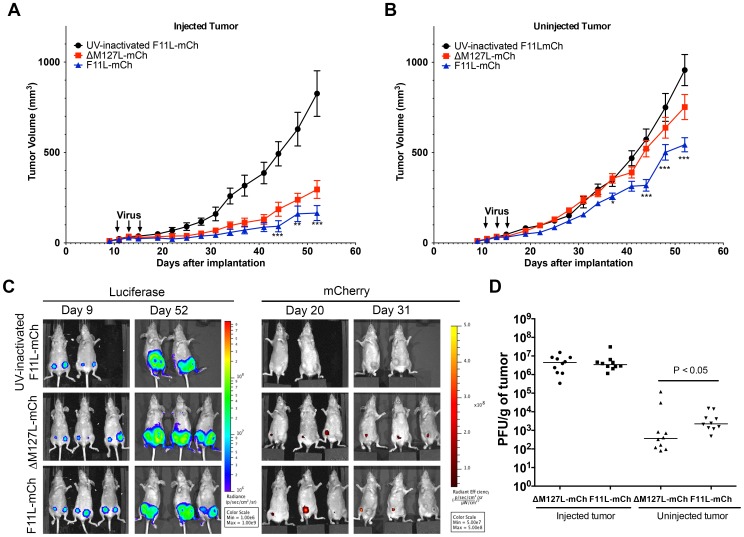
Effect of MYXV F11L-mCh on the growth of secondary untreated tumors. Two tumors (in opposite mammary fat pads) were established in NIH-III mice using MDA-MB-231 cells. Once palpable, the right-side tumor was injected with three doses of 5x10^7^pfu of ΔM127L-mCh (n = 10), F11L-mCh (n = 10), or an equivalent amount of UV-inactivated F11L-mCh virus (n = 8), and the tumor growth was monitored using calipers, with periodic imaging. After 6 weeks (experiment day 52) the mice were euthanized and the tissues titered to assay for virus. **(A)** Growth of tumors injected with MYXV. Calipers were used to monitor the growth of the tumors injected with the indicated viruses. **(B)** Growth of the uninjected contralateral tumor. In both (A) and (B) we report the mean tumor volume ± S.E.M and the statistics compare the cohorts treated with live ΔM127L-mCh or F11L-mCh viruses. **(C)** Tumor and virus imaging. An IVIS imager was used to detect tumor-encoded luciferase and virus- encoded mCherry on the days indicated. **(D)** Virus titers in excised tumors. Tumour tissues were recovered from the mice at the experimental endpoint, weighed, and the viruses titered on BGMK cells. The horizontal line denotes the mean titer.

Lastly, we wanted to test whether the superior tumor control obtained using the F11L-mCh virus would be restricted to the injected tumor. In particular, can F11L^+^ MYXV spread more efficiently to a second tumor site, one that had not been directly injected with virus? To investigate this question, we established bilateral tumors in opposite mammary fat pads. Once the tumors were palpable, the right-side tumor was injected with three doses of virus (5×10^7^ pfu/dose), while the left-side tumor was untreated. The size of both tumors was then monitored over a 6-week period, after which the mice were euthanized and the tumors and organs assayed for virus by plaque assay. The rate of growth of the tumors injected with live F11L-mCh virus was again slower than that in animals treated with the ΔM127L-mCh control virus. By the end of the experiment (52 days post-implantation) the animals treated with live F11L-mCh virus exhibited a mean tumor volume of 170±40 mm^3^ *versus* 300±50 mm^3^ in animals treated with live control virus (P<0.001). In comparison, mice treated with UV-inactivated virus had tumor volumes of 830±130 mm^3^ (**Figure 5A**).

Treating mice with live ΔM127L-mCh or F11L-mCh virus also inhibited the growth of the uninjected tumors. More interestingly, we observed that in mice injected with F11L-mCh virus, the untreated tumors at the conclusion of the experiment were significantly smaller than the untreated tumors in animals receiving ΔM127L-mCh virus (540±40 mm^3^ *versus* 750±70 mm^3^; P<0.001). In comparison, the untreated tumors in mice injected with UV-inactivated virus had grown by this point to a mean tumor volume of 960±90 mm^3^ (**Figure 5B**).

We were unable to use mCherry fluorescent signal to track virus distribution in the bilateral tumor model. In one instance a tumor injected with F11L^+^ virus was eradicated and a mCherry signal became detectable in the second untreated tumor. However, for the most part, the mCherry signal was obscured in the uninjected contralateral tumor. This detection may have been confounded by the fact the more intense mCherry signal in the injected tumors limited the IVIS imager from detecting any, presumably less intense signal, in the uninjected one. We also noted that near the end of the experiment (i.e. when mCherry signal would be more likely to be detected in the uninjected tumor) scabs formed at some of the tumor sites, which autofluoresced at wavelengths used to detect mCherry, and may have further confounded viral detection (**Figure 5C** **and data not shown**).

Nonetheless, virus production was confirmed by plaque assay at the end of the 6-week experimental endpoint. Similar levels of virus (∼4×10^6^ pfu/g tissue) were detected in the injected tumors in the two live virus treated groups, with several logs less virus detected in the untreated tumors. However, there was significantly (∼10-fold) more F11L-mCh virus detected in the uninjected tumors than in animals treated with the ΔM127L-mCh virus (**Figure 5D**). We also assayed other organs (brains, lungs, liver, spleen and kidneys), but could find no evidence of MXYV. The single exception was where we detected ∼300 pfu of virus in the lung of a mouse that had been treated with live F11L-mCh virus. This tissue contained no luciferase signal detectable by either IVIS imaging or western blot analysis, so whether this reflects trace virus contamination, virus spread to normal tissues, or a small amount of MYXV replicating in a small metastatic tumor nodule is difficult to determine.

## Discussion

At least two previous studies have shown that the effectiveness of oncolytic viruses is modulated by factors affecting viral dissemination. For example, reovirus capsid mutants have been identified that enhance plaque size in culture, and these mutant viruses appear to more effectively control B16 melanoma growth in C57BL/6 mice [29]. A mutation in the VACV A34 protein, which like F11, plays a role in proper EV release and VACV spread, has also been shown to increase VACV oncolytic efficacy in mouse tumor models, including CMT64 lung cancer, M38 colon cancer, and JC or 4T1 breast cancer [27], [28].

Despite showing promise as an oncolytic agent in several experimental systems, one factor that may limit using MYXV may be the low growth yields and the limited capacity to spread efficiently in hosts other than *Leporidae*. Here we showed that the growth and spread of MYXV can be improved by enhancing its capacity to disrupt actin-based cytoskeletal structures. This effect can be demonstrate in tissue culture using either pharmacological inhibitors, siRNA-mediated silencing of genes regulating the structure of the actin cytoskeleton, or by transgenic expression of the VACV F11L gene.

VACV F11 works by binding to, and disrupting, proteins that regulate the composition of actin-based structures [21], [24], [25]. This disrupts the integrity of the cortical actin layer, which facilitates the release of EV, and these EV are then thought to play an important role in creating the viremia that is a characteristic feature of VACV infections [27], [37]. This model is well supported by the observation that F11L-deficient VACV mutants spread less efficiently in infected mice [21]. Interestingly, while mutations that decrease the formation of EV attenuate VACV, viruses encoding mutations that increase the production of EV are no more pathogenic than those encoding WT alleles [27], [38]. This suggests that VACV may already be exploiting EV production systems for maximal benefit. There may still exist further gains to be obtained through the manipulation of these systems in MYXV using other kinds of recombinants, given that our F11L^+^ strain still doesn't produce nearly the same numbers of actin projectiles as does WT VACV [11].

The F11-mediated enhanced spread of MYXV also translated into better tumor control and prolonged survival of animals bearing xenografted human tumors. F11L+ MYXV also spread more efficiently to an uninjected contralateral tumor, with these tumors showing slower growth rates and higher levels of virus. While this virus appeared to spread better even at the highest doses we could deliver (three 50 µl injections of 5×10^7^ pfu), no toxicity was observed suggesting that this virus is safe, and at least in mice did not alter tropism. This is supported by observations that the introduction of F11L into the highly mutated MVA strain of VACV (which lacks a functional copy of F11L) was not sufficient to generate the tropism displayed by wild-type VACV[39]. In fact it would be highly unlikely that genes like F11L could alter the host range of other poxviruses, which is determined primarily by events occurring shortly after entry [[40]].

Our observation that F11 mediated improved growth of MYXV in a number of other types of cancer cells, suggests that this virus may have more widespread utility as an oncolytic agent. Furthermore, while these studies examined the oncolytic efficacy of F11L+ MYXV in an immunocompromised model, this modification may improve activity in an immunocompetent host, as this virus presumably releases more EV (**Figure 1F**) and EV produced in VACV infections are better capable of evading the immune system [41].

The enhanced growth of F11L^+^ MYXV appears to be linked to F11s to disruption of actin-based structures by inhibition of RhoA-signaling. Furthermore we note that the growth of wild-type MYXV could be enhanced by the treatment of cells with low concentrations of latrunculin B, or through siRNA-mediated silencing of RhoA, as well as RhoC, mDia1, or LIMK2. Interestingly, these treatments did not enhance production of the F11L^+^ MYXV strain, suggesting that these pathways can be saturated using disruptive interventions, beyond which the virus gains no additional advantages.

The up-regulation and activation of RhoGTPases, or their downstream effectors, has been linked with a number of processes critical to cancer progression including cell-proliferation, cell migration, and invasion, and has been the subject of a number of reviews [34], [35], [42], [43]. As such, it is widely recognized that elements of this pathway might be useful targets for cancer therapeutics (reviewed in [44]). Indeed, a number of inhibitors against proteins encoded by genes like RhoA, RhoC, mDia1, or LIMK2 are in various states of development [36], [44]–[47]. While we focussed on improving the oncolytic efficacy of MYXV primarily through genetic approaches, our work suggests that it may be possible to synergize these new chemotherapeutics with oncolytic virotherapy. Moreover, given that all viruses must overcome the challenge to exit posed by structures like cortical actin layer, and that cancer progression could potentially increase these barriers, this approach may be applicable to increasing the efficacy of other oncolytic viruses. Further work should investigate these possibilities.

## Materials and Methods

### Virus strains, cell-lines, and viral yield assays

The viruses used in these experiments have been previously described [11], [48]. All viruses encode β-galactosidase (vMYXlac) [48]. The ΔM127L-mCh and F11L-mCh viruses also express mCherry fluorescent protein under control of a synthetic early/late hybrid poxvirus promoter. Virus was purified by sucrose gradients as previously described [49], and resuspended in PBS. UV-inactivated virus stocks were generated by exposing virus to a UV lamp for 2h at a dose-rate of ∼7.5 J/sec/m^2^. Inactivation was confirmed by plaque assay.

BGMK cells were obtained from Diagnostic Hybrids (Athens OH, USA) and luciferase-expressing MDA-MB-231 cells (clone D3H2LN) were purchased from Caliper Life Sciences (Woodbridge ON, Canada). All other cell lines used in this paper were obtained from the American Type Culture Collection (Manassas VA, USA). The U87-MG, Daoy, MDA-MB-231 and BGMK cells were grown in minimal essential media (MEM) and MDA-MB-468 cells were propagated in 1∶1 DMEM/F12. All other cell lines were propagated in DMEM with high glucose. All of the cells used in this study were cultured with 10% fetal bovine serum (FBS), in the presence of 1% L-Glutamine, 1% non-essential amino acids, and 1% antibiotic/antimycotic. All cells used tested negative for mycoplasma by a Sigma PCR-based assay (Oakville ON, Canada).

Virus growth was determined by infecting sub-confluent 60 mm dishes of cells at a MOI of 0.01 for 1 h, before adding media. Virus was harvested at the indicated times and released from cells by three cycles of freeze-thaw before being titered on BGMK cells as previously described [11]. To measure the proportion of virus released into the media of infected cells, cells were infected at a MOI of 5 for 1 h. The inoculum was removed, cells washed with PBS, then fresh media added. After 24 h the medium was collected, centrifuged for 5 min at 1000×g, and virus was titered to quantitate released virus. Cell-associated virus was quantified by scraping the infected cell monolayer in PBS, releasing virus by three freeze-thaw cycles, and then titering on BGMK cells. These values were then used to calculate the percent of the total virus in the medium.

To measure cell viability following virus infection, 96 well dishes of cells were infected at the indicated MOI. After 96 h alamar blue (Sigma, Oakville ON Canada) was added to a concentration of 44 µM. After 2 h at 37°C, fluorescence was read using a Fluorstar Optima plate reader (544 nm excitation and 590 nm emission filters). Signal was then normalized to a percent of uninfected controls.

### Western blotting

Cells were washed with PBS, lysed, and the protein concentration determined (Biorad). Lysates were fractionated on SDS-PAGE gels, transferred to nitrocellulose, and probed with antibody. Antibodies used are as follows: RhoA (sc-4180), RhoC (D40E4), mDia1 (sc-10886), LIMK2 (sc-5577), ROCK1 (sc-17794) Ezrin (sc-58758) Cofilin (sc-33779), mCherry (632475), β-actin (A5441), M-T4[50], and F11[51]. M-T4 and F11 antibodies were gifts from Drs M. Barry and R. Condit. The RhoC antibody was purchased from Cell Signaling (Whitby, ON Canada) mCherry antibody from Clontech (Mountain View CA,USA) and β-actin antibody from Sigma. All other antibodies were purchased from Santa-Cruz Biotechnology (Dallas TX, USA). In general antibodies were used at manufacturers recommended concentrations. Infrared-conjugated secondary antibodies (Licor) and a Licor Odyssey imager (Lincoln NE, USA) were used to detect antibody-antigen complexes. β-actin served as a loading control.

### Microscopy

Fluorescence microscopy was performed as previously described [11]. The cells were fixed with 4% paraformaldehyde in PBS, blocked and then permeabilized using PBS-T. The cells were then stained with 0.3 U/mL Alexa Fluor 488-conjugated phalloidin and 5 ng/mL DAPI, to visualize actin and DNA, respectively. Cells were imaged using a DeltaVision microscope with either 20×(N.A. = 0.75) or 60×(N.A. = 1.42) objectives. Images were then processed using Softworx (v4.1.2). Micrographs of 50 cells from three separate experiments (i.e. n = 150) were used to quantify either actin stress fibres (8hpi), cell area (8hpi) or actin projectiles (20hpi) in an unblinded fashion. ImageJ software version 1.44i (http://rsbweb.nih.gov/ij/) was used to outline cells and determine cell area. To examine plaque differences (Figure 3B) cells were infected at MOI of 0.1, and fixed at stained with X-Gal 96 h later. Cells were imaged with a 10×objective (N.A. = 0.30) using a Zeiss Axioskop 2 microscope.

### Chemical inhibitors and siRNA experiments

Dharmafect 4 (Dharmacon) and antibiotic-free media was used to transfect MDA-MB-231 cells with 10 nM siRNA (Qiagen) in 6-well dishes. siRNAs used are: RhoA [hsRhoA-6 and hsRhoA-7], RhoC [hsRhoC-5 and hsRhoC-6] LIMK2 [hsLIMK2-7 and hsLIMK2-8], mDia1 [hsDIAPH1-1 and hsDIAPH1-3], or AllStars negative control siRNA. The cells were infected two-days later at MOI = 0.1 (∼10^4^ PFU/mL), cultured 48 h, harvested and titered. Alternatively the cells were infected and then treated with Y-27632 or latrunculin B (both from Sigma) at 4 or 8 hpi, respectively. The cells were harvested at 20 h for microscopy or 48 h to measure virus yield.

### Animal studies

Six to eight week old female NIH-III mice (Crl: NIH-*Lyst^bg-J^ Foxni^nu^ Btk^xid^*) were purchased from Charles River (Wilmington MA,USA) Tumors were established by injecting the mammary fat pad with 2×10^6^ MDA-MB-231 cells that had been mixed 1∶1 with Matrigel (BD Biosciences). Tumors became palpable 10–14 days later and were injected with virus when the volume reached 20–50mm^3^. For the single tumor model, three doses of virus (1×10^6^ pfu/dose) were intratumorally administered. Tumor volumes were measured twice weekly with calipers, and mice were euthanized when tumors reached 1500mm^3^.

For the bilateral tumor model, tumors were established in opposite mammary fat pads using the same method. Once tumors were palpable, the right side tumor was injected with three doses of virus (5×10^7^ pfu/dose) and tumors monitored for 6 weeks, after which mice were euthanized and tumors evaluated for virus levels. All studies were reviewed and approved by University of Alberta committees according to guidelines issued by the Canadian Council on Animal Care (http://www.ccac.ca/en).

An IVIS Spectrum imager was used to detect bioluminescence (luciferase) and fluorescence (mCherry) in infected mice. Luciferase activity was imaged 5–10 mins after intraperitoneal injection with 150mg/kg of D-luciferin (Gold Biotechnology). Virus-encoded mCherry signal was detected using 570nm and 680nm emission settings. The radiant efficiency was normalized to tumor area and is reported as [photons/s]/[µW/cm^2^].

### Statistics

Statistical analyses were performed using GraphPad Prism software (v.5). ANOVA tests with either one-way post-Tukey column comparisons, or two-way Bonferroni post-tests, were used to compare data comprising multiple viruses or time points. Paired T-tests were used to compare one siRNA treatment with another. Survival curve comparisons were calculated using the Log-Rank (Mantel-Cox) test built into Prism's software. P<0.05 were considered significant and statistically significant results reported as: * P<0.05, ** P<0.01, and *** P<0.001.

## Supporting Information

Figure S1 **Effect of 0.1 µM latrunculin B on actin structures in MYXV-infected MDA-MB-231 cells.** MDA-MB-231 cells were grown to sub-confluency on glass coverslips and then infected with the indicated viruses at MOI of 10. The cells were cultured for 8 h, the medium replaced with fresh medium containing 0.1 µM or the DMSO solvent, and the cells incubated for another 12 h and fixed. The cells were then stained with AlexaFluor 488-phalloidin and DAPI, to visualize actin and DNA, respectively, and fluorescence images obtained at 20× magnification. **(a)** Fluorescence microscopy showing cells infected with the different viruses in the presence or absence of latrunculin A. Scale bar  = 30 µm **(b)** Quantification of actin projectiles from Panel A. The graph shows the mean percentage of cells (±S.E.M) exhibiting 0, 1–2, or ≥3 actin projectiles per cell. The results were compiled from three independent experiments, analyzing 50 cells per experiment (*i.e.* n = 150).
(TIF)

Figure S2 **Effect of Y-27632 on actin structures in MYXV-infected MDA-MB-231 cells.** MDA-MB-231 cells were cultured and infected with MYXV as described above. Four hours later the medium was replaced with fresh medium containing 5 µM Y-27632, or no drug supplement, and the cells infected for another 16 h, fixed, and stained with AlexaFluor 488-phalloidin or DAPI. Scale bar = 30 µm **(a)** Fluorescence microscopy images showing infected cells at 20× magnification. **(b)** Quantification of actin projectiles from Panel a. The graph shows the mean percentage of cells (±S.E.M) exhibiting 0, 1-2, or ≥3 actin projectiles per cell. The results were compiled from three independent experiments, analyzing 50 cells per experiment (*i.e.* n = 150).
(TIF)

Figure S3 **Effect of F11 expression on MYXV growth in cancer cell lines under low MOI multi-step growth curve conditions.** Cancer cells were infected with respective viruses at a MOI of 0.01. At indicated times virus was harvested and titered on BGMK cells. The mean titer ± S.E.M., as normalized to PFU/10^6^ cells, from three independent experiments are shown. Data from the 72 h post-infection was used to generate Figure 3A.
(TIF)

Figure S4 **Effect of F11 expression on cell-viability of cancer cells infected with MYXV.** Cells were infected at the indicated MOI, with each of three different virus strains, in 96-well plates. The cells were cultured for 96 h, and the viability determined using Alamar blue dye. Viability is expressed as a percentage of that measured in uninfected cells. Mean cell viability as a percent ± S.E.M. from three independent experiments are reported. For comparison purposes data from the MDA-MB-231 cells is reproduced from Figure 1G.
(TIF)

Figure S5 **Western blot analysis of cellular proteins linked to regulation of the actin cytoskeleton.** **(a)** Schematic depicting the RhoA signaling pathway that leads to stress fiber formation and microtubule stabilization. Adapted and modified from [52] and [22] **(b)** Western blot analysis of cancer cell lines. The cells indicated were grown to sub-confluency in the absence of virus, harvested, lysed, and 20 µg of total protein separated using SDS-PAGE gels. Western blotting and infrared imaging was then used to measure the levels of the indicated proteins. The figure also shows the mean fold differences in virus yield at 72 h (±S.E.M.) when F11L-mCh and ΔM127L-mCh were grown on each cell line. These values were calculated from data presented in Figure 3.
(TIF)
