# Supplementary figures and images for: Myxoma Virus Oncolytic Efficiency Can Be Enhanced Through Chemical or Genetic Disruption of the Actin Cytoskeleton
Source: PLoS One. 2013 Dec 31;8(12):e84134. doi: 10.1371/journal.pone.0084134 (PMC3877188; doi:10.1371/journal.pone.0084134)

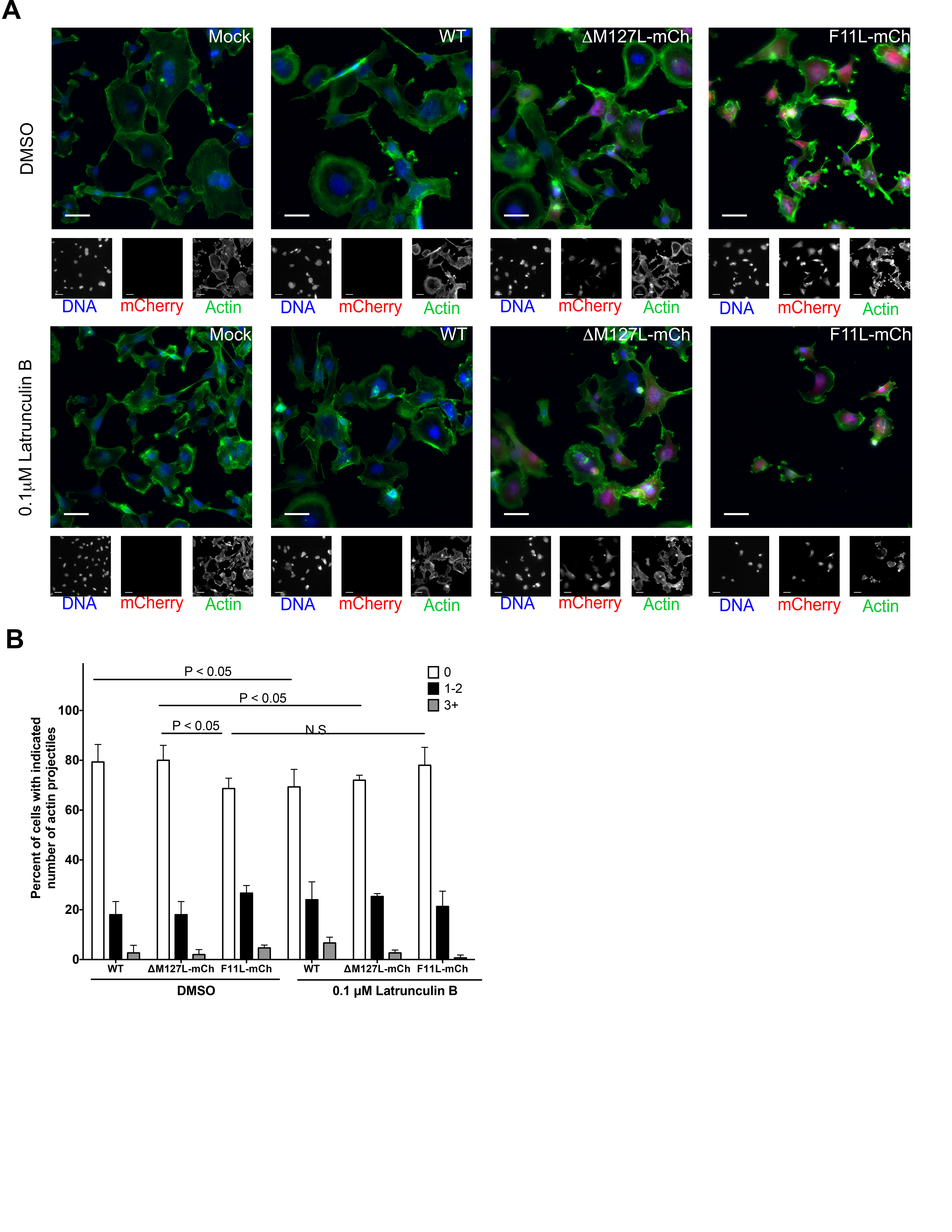

Supplement: Figure S1 — Effect of 0.1 µM latrunculin B on actin structures in MYXV-infected MDA-MB-231 cells. MDA-MB-231 cells were grown to sub-confluency on glass coverslips and then infected with the indicated viruses at MOI of 10. The cells were cultured for 8 h, the medium replaced with fresh medium containing 0.1 µM or the DMSO solvent, and the cells incubated for another 12 h and fixed. The cells were then stained with AlexaFluor 488-phalloidin and DAPI, to visualize actin and DNA, respectively, and fluorescence images obtained at 20× magnification. (a) Fluorescence microscopy showing cells infected with the different viruses in the presence or absence of latrunculin A. Scale bar = 30 µm (b) Quantification of actin projectiles from Panel A. The graph shows the mean percentage of cells (±S.E.M) exhibiting 0, 1–2, or ≥3 actin projectiles per cell. The results were compiled from three independent experiments, analyzing 50 cells per experiment (i.e. n = 150). (TIF) [file pone.0084134.s001.tif]

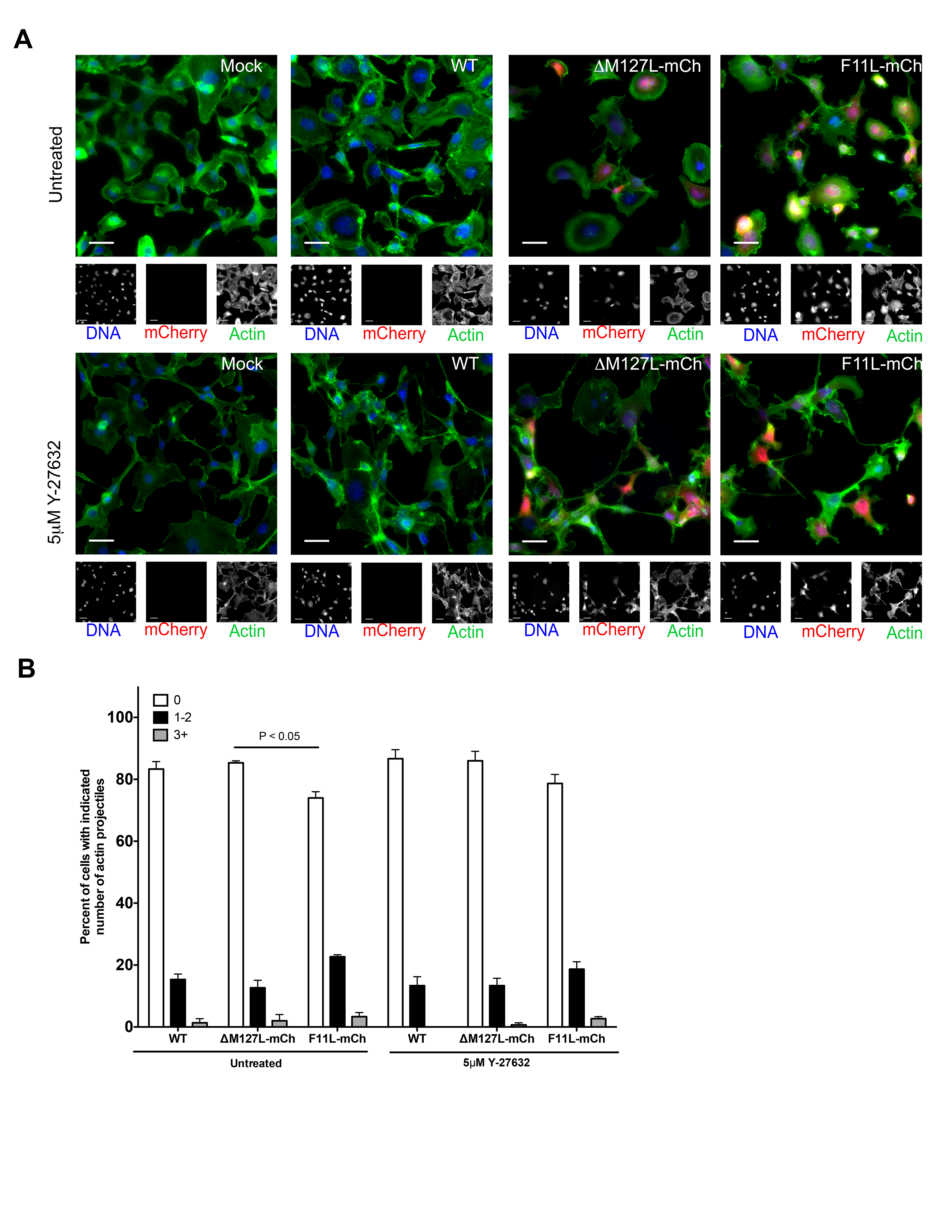

Supplement: Figure S2 — Effect of Y-27632 on actin structures in MYXV-infected MDA-MB-231 cells. MDA-MB-231 cells were cultured and infected with MYXV as described above. Four hours later the medium was replaced with fresh medium containing 5 µM Y-27632, or no drug supplement, and the cells infected for another 16 h, fixed, and stained with AlexaFluor 488-phalloidin or DAPI. Scale bar = 30 µm (a) Fluorescence microscopy images showing infected cells at 20× magnification. (b) Quantification of actin projectiles from Panel a. The graph shows the mean percentage of cells (±S.E.M) exhibiting 0, 1-2, or ≥3 actin projectiles per cell. The results were compiled from three independent experiments, analyzing 50 cells per experiment (i.e. n = 150). (TIF) [file pone.0084134.s002.tif]

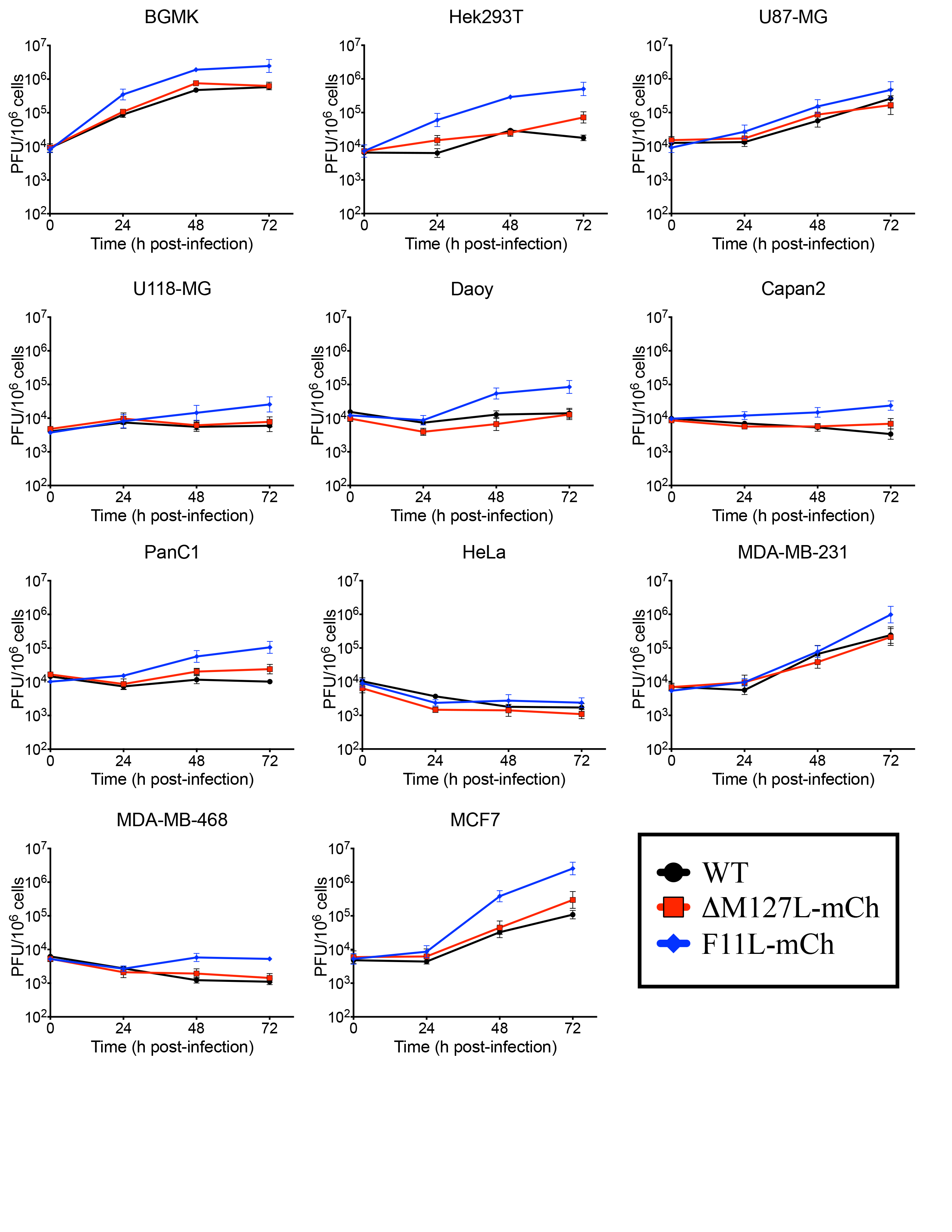

Supplement: Figure S3 — Effect of F11 expression on MYXV growth in cancer cell lines under low MOI multi-step growth curve conditions. Cancer cells were infected with respective viruses at a MOI of 0.01. At indicated times virus was harvested and titered on BGMK cells. The mean titer ± S.E.M., as normalized to PFU/106 cells, from three independent experiments are shown. Data from the 72 h post-infection was used to generate Figure 3A. (TIF) [file pone.0084134.s003.tif]

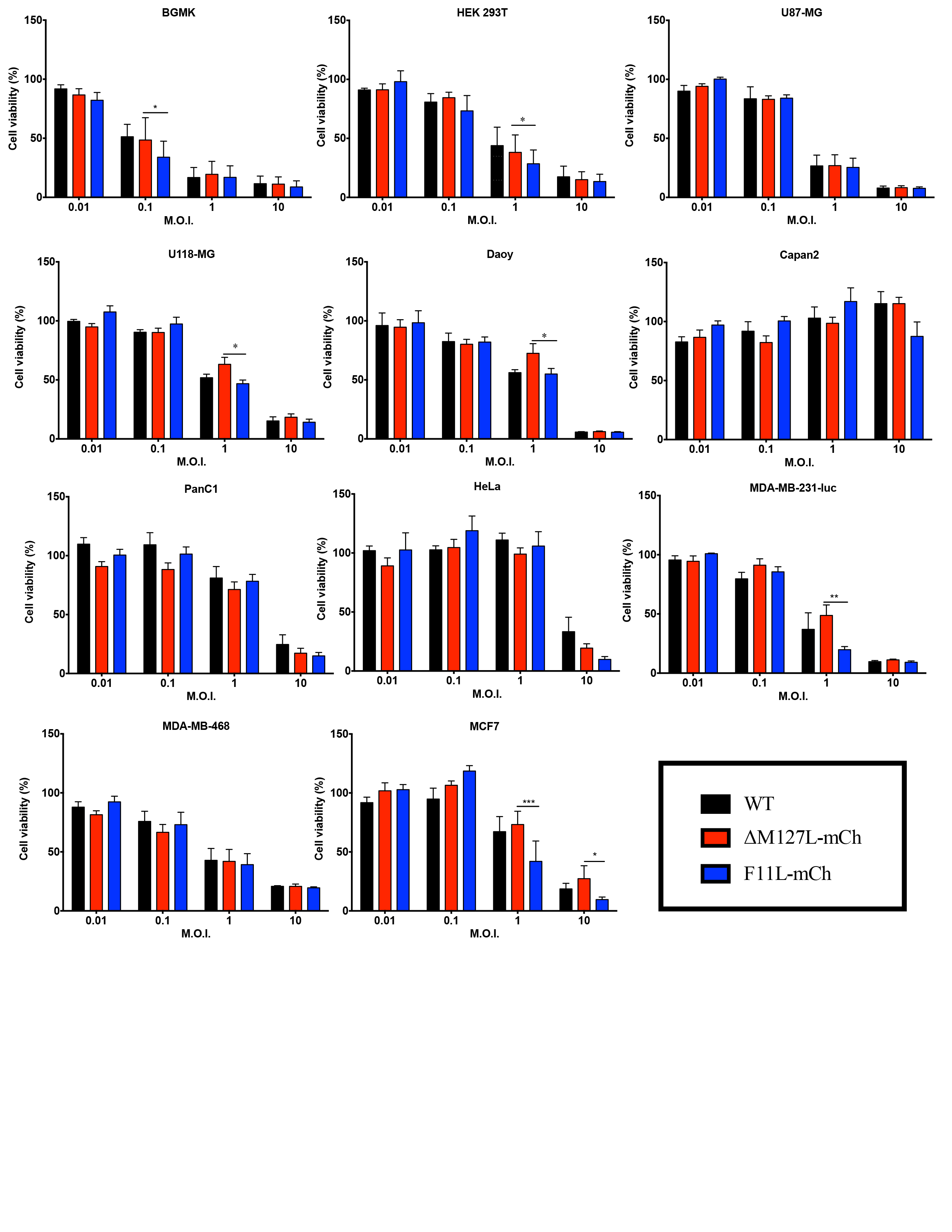

Supplement: Figure S4 — Effect of F11 expression on cell-viability of cancer cells infected with MYXV. Cells were infected at the indicated MOI, with each of three different virus strains, in 96-well plates. The cells were cultured for 96 h, and the viability determined using Alamar blue dye. Viability is expressed as a percentage of that measured in uninfected cells. Mean cell viability as a percent ± S.E.M. from three independent experiments are reported. For comparison purposes data from the MDA-MB-231 cells is reproduced from Figure 1G. (TIF) [file pone.0084134.s004.tif]

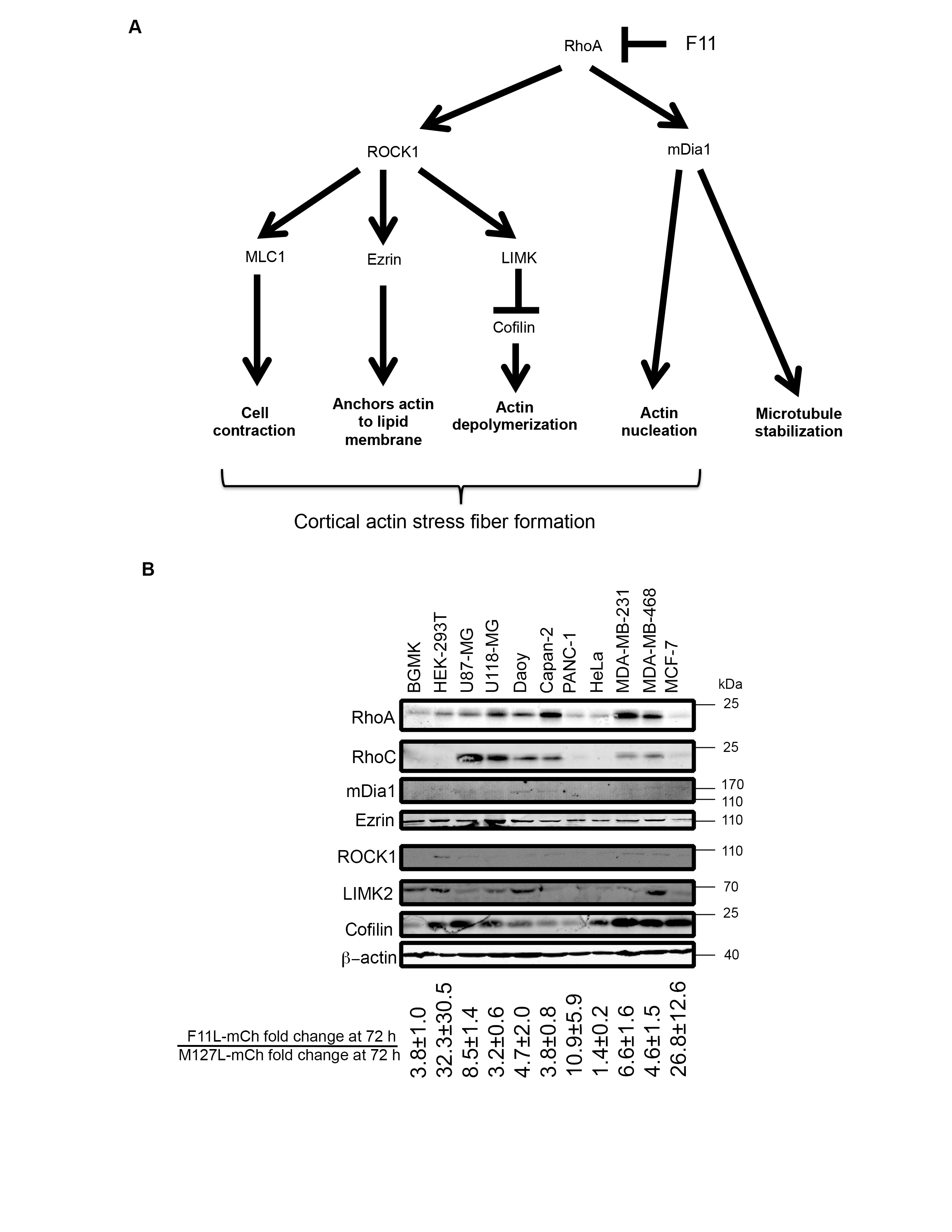

Supplement: Figure S5 — Western blot analysis of cellular proteins linked to regulation of the actin cytoskeleton. (a) Schematic depicting the RhoA signaling pathway that leads to stress fiber formation and microtubule stabilization. Adapted and modified from [52] and [22] (b) Western blot analysis of cancer cell lines. The cells indicated were grown to sub-confluency in the absence of virus, harvested, lysed, and 20 µg of total protein separated using SDS-PAGE gels. Western blotting and infrared imaging was then used to measure the levels of the indicated proteins. The figure also shows the mean fold differences in virus yield at 72 h (±S.E.M.) when F11L-mCh and ΔM127L-mCh were grown on each cell line. These values were calculated from data presented in Figure 3. (TIF) [file pone.0084134.s005.tif]
